# The Basis Substance in Calcified Products

**Published:** 1889-04

**Authors:** 


### Editorial.

### THE BASIS SUBSTANCE IN CALCIFIED PRODUCTS.

Your especial attention is called to the faithfulness with which the appearances seen in the tissues are dilineated in the plate in this issue. The illustrations were produced by a photographic process from the original photomicrographs made by Dr. Andrews. The reliability of the process is beyond question, and gives something that may be relied upon in that the illustration is the "shadow" outline of the phenomena presented in the original tissue, and not the result of a draughtsman's skill. You are requested to study the plate and draw your own conclusions in regard to the presentations there shown.

The formation of calcoglobulin has been the subject of investigation for many years, and the manner of its development is now very generally understood. In our writings upon the subject, which were published in the American System of Dentistry, we treated of the matter quite fully, dwelling more especially, however, upon the nature of the process than upon the phenomena observed, considering that the latter had been so well presented by former writers as to need no further elucidation. We have gone over the experiments and substantiated the correctness of the observations of Mr. Rainie, and in addition, studied the formation of renal, billiary and vesical calculi, where the menstruum, in many if not in a majority of instances, is composed of mucus or pus. We there pointed out the probable manner in which lime salts were deposited, under the superintendency of the living cells, in the intercellular protoplasm, giving rise to a new product, and indicated that the form of the resulting product was directly under the control of the life force, while the chemical nature of the tissue thus formed was not, in that a similar product could and had been produced in the laboratory without the intervention of the living principle found in nature.

The evidence that Dr. Andrews brings to our notice is of a different character, being an ocular demonstration of the peculiar conditions presented in the special tissues under consideration, and herein lies its value. Let us consider its special features, and in so doing we will state in the beginning, that we have carefully studied the original slides kindly furnished us by Dr. Andrews, and have discovered what seems to us to be the key to the explanation of the special phenomenon shown in the plate.

There can be no question that Dr. Andrews is correct in his conclusion that the globular masses seen on the border land of calcification are of the nature of calcoglobulin first described by Mr. Rainie, and afterwards brought forward by Mr. Tomes and ourselves, in discussing the subject. There is, however, a further lesson to be learned from the evidence.

Three questions arise in regard to the nature of the process herein presented, viz: as to whether the tissue delineated is normal, pathological, or the result of morphological changes that have taken place during the hardening process. If the condition were artificial, as some seem inclined to consider it, then we should expect to find the forming surface of the dentine throughout the entire specimen, presenting the same globular appearance, which is not the case, as was demonstrated by a careful study of the original specimens. In other portions of the sections the forming dentine showed the even, smooth gradation from organic to calcified tissue. This was so throughout all the sections examined. The particular condition, then, is local in character, and we must therefore dismiss the idea of its being the result of post-mortem change.

As opposed to the theory presented, that it is the normal production of the basis substance, we have to array our own experience and say that in our studies, extending over several years' constant work in embryology, that we have never met so marked a case of globular formation upon the border-land of calcification in any portion of the body as is here delineated. While we have seen a more or less constant tendency upon the part of forming calcified tissues to produce the small bodies denominated calcospherites, and which have also been observed by Messrs. Robin and Magitot in the pulps of developing human teeth, and by Henle in those of the herbivera, yet it has never been our fortune to observe the globular masses shown in the slides from which the plate was made.

This, however, may be said to be only negative testimony, nevertheless in the case in hand such evidence is of the greatest importance in establishing our view of the case, viz: that it is pathological in character for, a process to be considered normal must be constant in its presentation. The fact that at least one observer has failed to find it so is more or less conclusive evidence that it is not what might be termed a normal or physiological appearance. When we take into consideration that pathological processes are only perverted physiological processes, which may be only one step removed from the normal, it does not necessarily mean any great divergance from the normal process of development. Then again the same argument that was used against the the idea of its artificial nature can be here used, seeing that the other portions of the tissues in the same section do not present a similar character. Our interpretation of the phenomena presented in the plate is that through some cause, the nature of which we are unable to explain—there has been a local disturbance in the physiological process and these large (microscopically) globular bodies have resulted.

Mr. Tomes has described what we take to be similar appearances, and if we understand him correctly, holds the same view with us, when he says "that globular, spherical forms are constantly to be seen at the edge of the thin cap of forming dentine and may be traced in and around the interglobular spaces." In another place he says, that " although these spaces are very common, they are perhaps not to be regarded as perfectly normal, but are rather indications of an arrested development at that spot."

The above interpretation of the condition does not in any way detract from the value of the illustration but on the other hand, to our mind, enhances it in that it offers an explanation for the formation of " Interglobular spaces."

